# Novel Insights into Diagnosis, Biology, and Treatment of Primary Diffuse Leptomeningeal Melanomatosis

**DOI:** 10.3390/jpm11040292

**Published:** 2021-04-12

**Authors:** Alicia Baumgartner, Natalia Stepien, Lisa Mayr, Sibylle Madlener, Christian Dorfer, Maria T. Schmook, Tatjana Traub-Weidinger, Daniela Lötsch-Gojo, Dominik Kirchhofer, Dominik Reisinger, Cora Hedrich, Saleha Arshad, Stefan Irschik, Heidrun Boztug, Gernot Engstler, Marie Bernkopf, Fikret Rifatbegovic, Christoph Höller, Irene Slavc, Walter Berger, Leonhard Müllauer, Christine Haberler, Amedeo A. Azizi, Andreas Peyrl, Johannes Gojo

**Affiliations:** 1 Department of Pediatrics and Adolescent Medicine and Comprehensive Center for Pediatrics, Medical University of Vienna, 1090 Vienna, Austria; alicia-christina.baumgartner@meduniwien.ac.at (A.B.); natalia.stepien@meduniwien.ac.at (N.S.); lisa.mayr@meduniwien.ac.at (L.M.); sibylle.madlener@meduniwien.ac.at (S.M.); dominik.reisinger@meduniwien.ac.at (D.R.); cora.hedrich@meduniwien.ac.at (C.H.); saleha.arshad@meduniwien.ac.at (S.A.); stefan.irschik@meduniwien.ac.at (S.I.); irene.slavc@meduniwien.ac.at (I.S.); amedeo.azizi@meduniwien.ac.at (A.A.A.); andreas.peyrl@meduniwien.ac.at (A.P.); 2 Comprehensive Cancer Center-Central Nervous System Tumors Unit, Medical University of Vienna, 1090 Vienna, Austria; daniela.loetsch-gojo@meduniwien.ac.at (D.L.-G.); walter.berger@meduniwien.ac.at (W.B.); 3 Institute of Cancer Research, Department of Medicine I, Medical University of Vienna, 1090 Vienna, Austria; dominik.kirchhofer@meduniwien.ac.at; 4 Department of Neurosurgery, Medical University of Vienna, 1090 Vienna, Austria; christian.dorfer@meduniwien.ac.at; 5 Department of Biomedical Imaging and Image-Guided Therapy, Medical University of Vienna, 1090 Vienna, Austria; maria.schmook@meduniwien.ac.at (M.T.S.); tatjana.traub-weidinger@meduniwien.ac.at (T.T.-W.); 6 Department of Pediatric Hematology and Oncology, St. Anna Children’s Hospital, Medical University of Vienna, 1090 Vienna, Austria; heidrun.boztug@stanna.at (H.B.); gernot.engstler@stanna.at (G.E.); 7 Children’s Cancer Research Institute, 1090 Vienna, Austria; marie.bernkopf@ccri.at (M.B.); fikret.rifatbegovic@ccri.at (F.R.); 8 Department of Dermatology, Medical University of Vienna, 1090 Vienna, Austria; christoph.hoeller@meduniwien.ac.at; 9 Department of Pathology, Medical University of Vienna, 1090 Vienna, Austria; leonhard.muellauer@meduniwien.ac.at; 10 Division of Neuropathology and Neurochemistry, Department of Neurology, Medical University of Vienna, 1090 Vienna, Austria; christine.haberler@meduniwien.ac.at

**Keywords:** leptomeningeal melanomatosis, leptomeningeal melanocytosis, primary diffuse leptomeningeal melanomatosis, melanocytic tumors, targeted therapy, precision medicine, m-TOR inhibitor, MEK-inhibitor, PD-1-inhibitor, CTLA-4-inhibitor, liquid biopsy, disease monitoring

## Abstract

Primary diffuse leptomeningeal melanomatosis (PDLMM) is an extremely rare and aggressive cancer type for which best treatment strategies remain to be elucidated. Herein, we present current and prospective diagnostic strategies and treatment management of PDLMM. Against the background of an extensive literature review of published PDLMM cases and currently employed therapeutic strategies, we present an illustrative case of a pediatric patient suffering from PDLMM. We report the first case of a pediatric patient with PDLMM who received combination treatment including trametinib and everolimus, followed by intravenous nivolumab and ipilimumab with concomitant intensive intraventricular chemotherapy, resulting in temporary significant clinical improvement and overall survival of 7 months. Following this clinical experience, we performed a comprehensive literature review, identifying 26 additional cases. By these means, we provide insight into current knowledge on clinical and molecular characteristics of PDLMM. Analysis of these cases revealed that the unspecific clinical presentation, such as unrecognized increased intracranial pressure (present in 67%), is a frequent reason for the delay in diagnosis. Mortality remains substantial despite diverse therapeutic approaches with a median overall survival of 4 months from diagnosis. On the molecular level, to date, the only oncogenic driver reported so far is mutation of *NRAS* (*n* = 3), underlining a close biological relation to malignant melanoma and neurocutaneous melanosis. We further show, for the first time, that this somatic mutation can be exploited for cerebrospinal fluid liquid biopsy detection, revealing a novel potential biomarker for diagnosis and monitoring of PDLMM. Last, we use a unique patient derived PDLMM cell model to provide first insights into in vitro drug sensitivities. In summary, we provide future diagnostic and therapeutic guidance for PDLMM and first insights into the use of liquid biopsy and in vitro models for this orphan cancer type.

## 1. Introduction

Primary diffuse leptomeningeal melanomatosis (PDLMM), a primary meningeal melanocytic tumor, is an extremely rare and highly aggressive disease for which standardized treatment and cure are still lacking. Since only 26 cases have been reported in the literature so far, estimation of incidence is currently not possible and both clinical and biological knowledge about this disease is scarce. 

Primary central nervous system (CNS) melanocytic tumors are neoplasms originating from melanocytes, which are derived from the neural crest and also migrate to the leptomeninges during embryonic development. According to the 2016 WHO classification, melanoblasts can give rise to four groups of neoplasms: the circumscribed melanocytoma (benign) or melanoma (malignant) and the primary diffuse leptomeningeal melanocytosis (PDLMC, benign) or melanomatosis (PDLMM, malignant), which also exhibit distinct molecular profiles [1,2]. Nevertheless, brain metastases of malignant melanoma (MM) are more frequent than all of these entities together, highlighting the importance of studies exploring the translational potential of knowledge based on MM research to melanocytic CNS tumors. 

MM ranks among the malignancies with the highest mutational burden and is classified into four genomic groups based on the presence of distinct oncogenic drivers: mutant *BRAF* (50% of all melanomas), mutant *N-/K-/H-RAS* (25%), mutant *NF1* (15%), and triple-wildtype (5%) [3,4]. Of note, at least *BRAF* and *NRAS* mutations are also found in benign naevi, highlighting the importance of additional somatic mutations, which are often found in *CDKN2A*, *PTEN*, *TERT* promoter or *TP53* [4,5].

On the cell biological level, these oncogenic mutations of *RAS* or *BRAF* lead to aberrant activation of the mitogen-activated kinase (MAPK) pathway [6]. In addition, mutated *N-RAS* activates the phosphatidylinositol-3 (PI3K) pathway [7], which triggers activation of the serin/threonine kinase V-Akt Murine Thymoma Viral Oncogene Homolog (AKT) and, subsequently, Mechanistic Target Of Rapamycin Kinase (mTOR) and Ribosomal Protein S6 (S6), another important regulator of cell growth, proliferation, and survival [8]. Thus, it is of utmost importance to explore whether these pathways are also activated in PDLMM and how much of the scientific and therapeutic progress achieved in the field of MM can be applied to PDLMM.

With respect to clinical management, early diagnosis of PDLMM is highly challenging since symptoms and findings on imaging are often unspecific, and final diagnosis of PDLMM is a diagnosis of exclusion. The definitive diagnosis needs to be confirmed by histopathological tissue analysis. This often proves to be difficult due to the risk of obtaining false negative biopsies, on one hand, and inaccurate classification, on the other hand [9,10]. Pathologically, PDLMC and PDLMM are characterized by variably shaped tumor cells comprising round, ovoid, cuboidal, or spindle shaped forms, spread across the leptomeninges. Whereas in PDLMC tumor cells look bland and do not infiltrate the brain, in PDLMM, generally, marked cytological atypia and mitotic activity, as well as infiltration into the brain parenchyma, is present [2]. The latter qualifies for PDLMM even in the absence of nuclear atypia and lack of mitoses. However, the molecular differences and similarities of PDLMC and PDLMM have not yet been explored in greater depth.

As to our knowledge, no clinical trial has been performed in PDLMM, and guidelines for its management are lacking; treatment (including surgery, chemotherapy, and radiation) for PDLMM has so far been performed based on the knowledge available for leptomeningeal metastases originating from extracranial MM. Because of the characteristic diffusely infiltrating tumor growth, gross total resection is impossible. Moreover, PDLMM frequently exhibits resistance to radio- and chemotherapy [10,11,12,13]. As a consequence, the prognosis is considered extremely poor [14].

Nevertheless, recent advances in molecular genetics and the development of targeted therapies being successfully applied in malignant melanoma raise the hope for more effective therapies for PDLMM. The combination of B-Raf Proto-Oncogene (BRAF)- and Mitogen-Activated Protein Kinase Kinase 1 (MEK)-inhibitors has shown an important improvement in the prognosis of *BRAF-V600E* mutant MM cases, while immunotherapy with check-point inhibitors, like Programmed Cell Death 1 (PD-1) and/or Cytotoxic T-Lymphocyte Associated Protein 4 (CLTA-4) blocking antibodies, has shown activity in melanoma patients irrespective of their mutational status and is currently the recommended first-line therapy [15]. While MEK-inhibitors as monotherapy have been tested in *NRAS* mutant melanoma patients and have shown a minor advantage in progression free survival (PFS) without an impact on overall survival [16], they are still used in selected cases lacking alternative treatment options and result in additional benefit in selected cases [17]. It remains to be elucidated whether one of these therapeutic strategies in MM may be a suitable therapy option for PDLMM. 

Against this background, we present a rare case of pediatric primary diffuse leptomeningeal melanomatosis, discuss the therapeutic approach, and provide a comprehensive analysis of clinical course and molecular characteristics of PDLMM. Moreover, we give first in vitro evidence of potential anti-cancer therapy and explore the potential of liquid biopsies in order to improve the outcome of this particularly aggressive form of CNS malignant melanoma by future precision medicine approaches.

## 2. Materials and Methods

### 2.1. Patient Material and Ethical Considerations

Patient material was obtained from neurosurgical procedures and therapeutic interventions performed at the Medical University of Vienna. Informed consent was obtained and the study was approved by the institutional review board of the Medical University of Vienna (EK Nr. 1244/2016). 

### 2.2. Case Study

In this study, we report on a single case of a pediatric patient with PDLMM being treated at our department. The case study was performed by detailed in-depth data collection using multiple sources of information, including print, as well as digital patient charts, clinical findings, laboratory values, radiological imaging, and histopathological, as well as molecular, analyses.

### 2.3. Literature Review

A systematic PubMed and Embase search were performed using the keywords “‘leptomeningeal melanomatosis’/exp OR ‘leptomeningeal melanomatosis’ OR (leptomeningeal AND (‘melanomatosis’/exp OR melanomatosis))”. Reports were included for patients of all ages with primary diffuse leptomeningeal melanomatosis without evidence of extracranial primary tumor, neurocutaneous melanosis, or large/giant congenital nevi. Cases with one nodular mass as opposed to the characteristic diffuse leptomeningeal infiltration were excluded. Clinical characteristics, therapeutic strategies, genetic alterations, and outcome were extracted and compared to our case. This review yielded an additional 26 cases classified as primary diffuse leptomeningeal melanomatosis. The flow chart of the review is depicted in Supplementary Materials Figure S1 and summarized in Supplementary Materials Table S1.

### 2.4. Neuropathology

Cerebrospinal fluid (CSF) was routinely processed and cytospin preparations were prepared and stained with May Grünwald-Giemsa (MGG). Neurosurgical biopsy specimens were formalin fixed, paraffin-embedded, and 3-µm thick sections stained with hematoxylin and eosin (HE). For immunohistochemical stainings, the following antibodies were used: anti-Melanosome (Clone HMB-45, Dako Agilent, Santa Clara, CA, USA, dilution 1:50) and anti-PD-L1 (clone BSR 90; BioSite, Täby, Sweden, dilution 1.50). 3-Amino-9-Ethylcarbazole AEC (Sigma-Aldrich, St. Louis, MO, USA) was used as chromogen to avoid confusion with melanin.

### 2.5. Next Generation Sequencing (NGS)

Cell-free tumor DNA (ctDNA) was isolated from CSF using the “Quick-cfDNA/RNA Serum & Plasma Kit” (Zymo Research, Irvine, CA, USA) according to the manufacturer’s instructions. NGS was performed on the biopsy tissue and on ctDNA with oncomine comprehensive assay v3 (Thermo Fisher Scientific, Waltham, MA, USA) according to the manufacturer’s instructions [18].

### 2.6. Cell Models

The primary leptomeningeal melanomatosis cell model VBT384 was derived from a biopsy at the Department of Neurosurgery, Medical University of Vienna. The *NRAS* mutant melanoma models VM9 and VM15 were established at the Institute of Cancer Research (Medical University of Vienna, as previously published [19,20]). The cell models were cultured in RPMI-1640 medium (Sigma-Aldrich, St. Louis, MO, USA) supplemented with 10% fetal calf serum (FCS, Gibco, Thermo Fisher Scientific, Waltham, MA, USA) and kept under humidified conditions containing 5% CO_2_ at 37 °C (normal cell culture conditions). Neither antibiotics nor any other anti-microbial substances were used during this study. 

#### 2.6.1. Adenosine triphosphate (ATP) Assay

Everolimus, trametinib, and palbociclib were purchased from Selleck Chemicals (Houston, TX, USA). VBT384 cells were plated in triplicates (4 × 10^4^ cells/mL) in 100 μL growth medium per well in 96-well plates. Following a 24 h recovery time under normal cell culture conditions, trametinib (0 to 10 μM), everolimus (0 to 50 µM), and palbociclib (0 to 10 µM) were added alone or in different combination regimens in 100 μL growth medium with 10% FCS, and cells were exposed for 72 h. The proportion of viable cells was determined by ATP assay following the manufacturer’s recommendations (“CellTiter-Glo^®^ Luminescent Cell Viability Assay”, Promega, Madison, WI, USA). Luminescence was measured at 1000 nm at the Tecan infinite 200Pro (Zurich, Switzerland). Raw data were analyzed using GraphPad Prism software 8.0 (GraphPad Software Inc., San Diego, CA, USA). Results are given as mean +/− SD and were normalized to untreated control cells. Cytotoxicity was expressed as IC_50_-values calculated from full dose-response curves (drug concentrations inducing a 50% reduction of the cell number in comparison to the untreated control cells). The interaction between the activities of combined drugs is expressed by the combination index (CI) as published by Chou [21] using CalcuSyn software (Biosoft, Ferguson, MO, USA). CI < 0.9, CI = 0.9–1.2 or CI > 1.2 represent synergism, additive effects and antagonism, respectively.

#### 2.6.2. Protein Isolation and Western Blotting

Total protein fractions were extracted from cells in confluent T25-flasks either under standard culture conditions or treated with a combination of 5 µM trametinib and 10 µM everolimus for six hours and processed for Western blotting on SDS-PAGE gels. Total protein concentrations were determined following manufacturer’s instructions (“Pierce™ BCA Protein Assay Kit”, Rockford, IL, USA). Antibodies detecting target proteins (Supplementary Materials Table S2) were diluted 1:1000 in 3% bovine serum albumin in Tris-buffered saline with 0.1% Tween20. Mouse or rabbitperoxidase-labeled secondary antibodies (Santa Cruz Biotechnology, Dallas, TX, USA) were used at working dilutions of 1:10000.

### 2.7. Liquid Biopsy

CSF samples were obtained via an external ventricular drain or an Ommaya reservoir, always directly preceding intraventricular chemotherapy. CSF was aliquoted into 1 ml vials and frozen at −80 °C within an hour of acquisition. Prior to analysis, samples were thawed and centrifuged, to eliminate cell debris. Cell-free DNA (cfDNA) was isolated using the “cfDNA/RNA Serum & Plasma Kit” (Zymo Research, Irvine, CA, USA) following the manufacturers instruction with minor modifications.

Due to the very small amounts of cfDNA, a preamplification step was performed using the Ssoadvanced™ preAmp Kit (Bio-Rad Laboratories Inc., Hercules, CA, USA). The following customized primers were used for PCR: forward: 5′-TTG AGA GTT GCT GCC CTA GC-3′; reverse: 5′-GTT CCA AAA GCC CCT CAG GA-3′.

Digital droplet PCR (ddPCR) was performed with a predesigned and validated probe from BioRad (Assay ID: dHsaMDS882187944) (Bio-Rad Laboratories Inc., Hercules, CA, USA) for *ƒ* using the amplified DNA samples. Mutation allele frequency was estimated using the following formula: (MAF = $\frac{total\;counts\;of\;MT+total\;counts\;of\;WT}{total\;counts\;of\;MT}$).

## 3. Results

### 3.1. Illustrative Case

**Figure 1:**
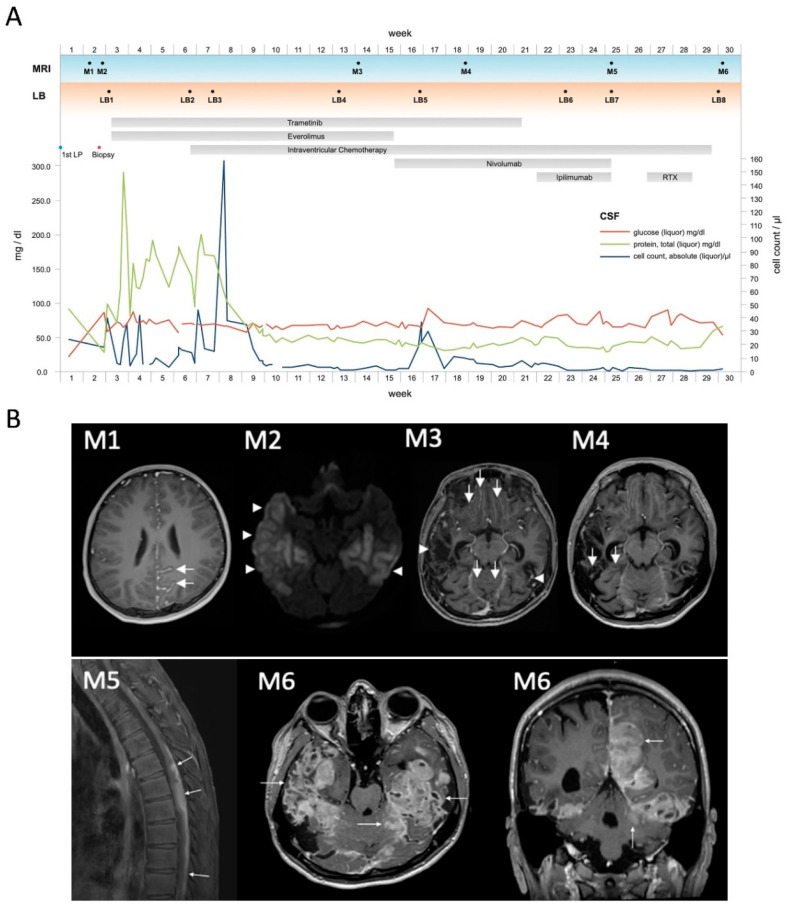
Illustrative case. (**A**) Time course of parameters evaluated in our patient in relation to significant clinical events. Parameters evaluated include CSF values, radiological studies and liquid biopsy results. CSF: cerebrospinal fluid; LB 1–8: timepoints of liquid biopsy; LP: lumbar puncture; RTX: radiotherapy; M1–M6: timepoints of magnetic resonance (MR) imaging exams. (**B**) M1, axial contrast enhanced (CE) T1-weighted MR image at diagnosis showing leptomeningeal contrast enhancement in the left parietooccipital region (arrows); M2, axial image of diffusion weighted imaging (DWI, b1000 map) depicting restricted diffusion in bilateral temporal ischemia (arrow heads) after biopsy; M3, axial contrast enhanced T1-weighted MR image in week 14 demonstrating leptomeningeal enhancement in frontobasal and cerebellar sulci (arrows). Note the postischemic parenchymal defects (arrow heads). M4, Axial CE T1-weighted MR image in week 18 showing progression of leptomeningeal enhancement in cerebellar sulci. M5, Sagittal T1-weighted contrast enhanced image with fat suppression demonstrating dorsally located perimedullary spread of the disease (white arrows) with infiltration of the thoracic spinal cord. M6, T1-weighted contrast enhanced images in axial and coronal plane showing progressive disease with large confluent leptomeningeal and superficial parenchymal lesions (arrows). Note that they are also occupying the postischemic defects in the temporobasal region.

**Figure 2:**
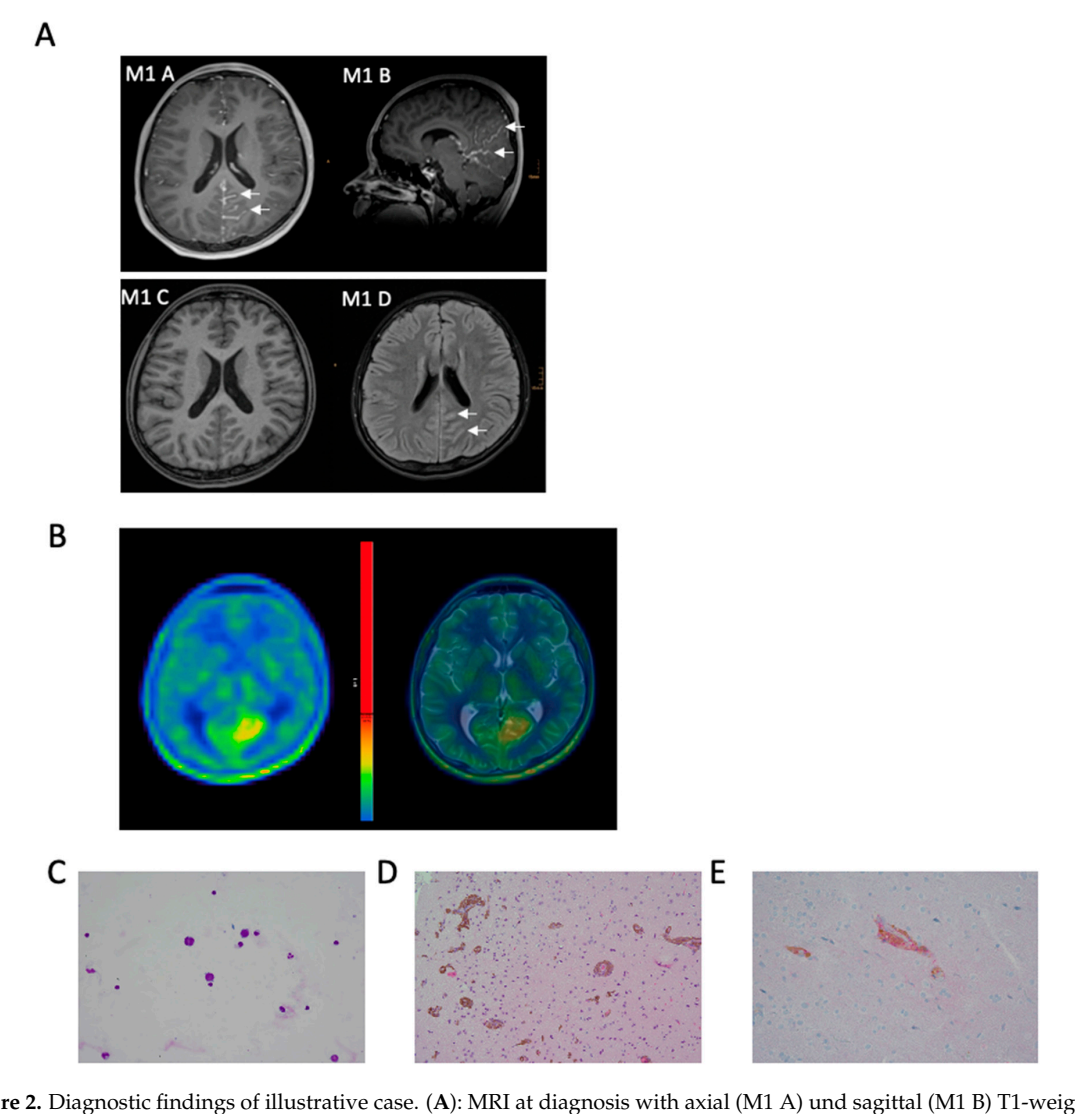
Diagnostic findings of illustrative case. (**A**): MRI at diagnosis with axial (M1 A) und sagittal (M1 B) T1-weighted contrast enhanced images showing leptomeningeal enhancement within parietooccipital sulci (arrows); corresponding native T1-weighted MR image (M1 C) showing no hyperintensities within the affected sulci; T2-weighted MR image with CSF suppression depicting incomplete CSF suppression within the affected sulci: (**B**): 11-C- methionine positron emission tomography (PET) and T2 axial, (**C**): numerous large tumor cells with round to ovoid nuclei and scant cytoplasm in CSF, (**D**): hematoxylin and eosin (HE) sections of the biopsy specimens revealed cortex with numerous perivascularly arranged sleeve-like aggregates of pigmented tumors cells characteristic for primary diffuse leptomeningeal melanomatosis (PDLMM), (**E**): tumor cells expressing membranous PD-L1.

In July 2020, a previously healthy 14-year-old boy was first treated at a peripheral hospital complaining about severe fluctuating headaches and vomiting upon waking up for the last two weeks. Following acute additional disorientation, a magnetic resonance imaging (MRI) scan of the head and spine was performed but showed no signs of malignancy or elevated intracranial pressure. An electroencephalogram (EEG) revealed slow background activity without seizure foci. A subsequent lumbar puncture (LP) (Figure 1A, 1st LP) disclosed numerous large tumor cells with round to ovoid nuclei and scant cytoplasm (Figure 2C) in the CSF, as well as elevated levels of protein and lactate and a reduced glucose concentration. The lumbar puncture led to an immediate improvement in clinical symptoms, indicative of increased intracranial pressure (ICP). Additionally, an ophthalmologic examination revealed bilateral papilledema.

On admission to the tertiary care center at the Medical University of Vienna the cranial MRI scan was repeated again without showing any solid mass. However, an increased cranial and spinal leptomeningeal gadolinium contrast enhancement was detected (Figure 1B, M1; Figure 2A). For further characterization a 11-C-methionine (Met) positron emission tomography (PET) combined with computed tomography (CT) was performed, demonstrating increased tracer uptake in the area of the left parietooccipital lobe (Figure 2B). Whole body fluorodeoxyglucose (FDG)-PET prior to biopsy did not show any increased uptake outside of the CNS. An open biopsy was performed within the area of maximum methionine uptake (left occipital leptomeningeal lesion). HE staining of the biopsy specimens revealed cortex tissue with numerous perivascularly arranged sleeve-like aggregates of pigmented tumors cells (Figure 2D, HE staining), which were HMB-45 immunoreactive. Molecular Immunology Borstel (MIB1) labeled tumor cells were absent. Membranous PD-L1 expression was detectable in some of the tumor cells (Figure 2E).

The histological findings, as well as the absence of lesions outside the CNS, led to the diagnosis of PDLMM three weeks after the onset of symptoms. 

Due to decreasing vigilance the patient had to be intubated and an extraventricular drain (EVD) and a cerebral pressure probe were placed. Gradually the clinical symptoms originating from the increased intracranial pressure improved. Postoperatively the patient experienced bilateral non-territorial temporal ischemia with cortical predominance which could not be unequivocally assigned to a specific vascular territory (Figure 1B, M2). It is worth noting that a microvascular disturbance due to the evident perivascular infiltration of tumor cells (Figure 2D) might have contributed to the observed ischemic events in the perioperative setting. Antiepileptic therapy with levetiracetam was initiated to prevent convulsions considering the increased susceptibility to seizures in the context of PDLMM. 

Based on the experience in the therapy of MM, the lack of established treatment strategies for PDLMM, and the high clinical need, we initiated treatment with oral trametinib (initially 2 mg 1 ×/day; after one week adjusted to 1.5 mg; 1 ×/day) and everolimus (7.5 mg 1 ×/day, adjusted according to serum target level of 5–15 ng/mL). After ten days, the patient was extubated without complications. In addition, we initiated intraventricular chemotherapy with etoposide (0.5 mg 2 ×/day administered via EVD). Following continuous clinical improvement, the patient could be transferred to the neuro-oncology ward after 23 days in the intensive care unit. Because of the improving clinical status and no signs of ICP, the EVD was exchanged for an Ommaya reservoir one month postoperatively. Afterwards, the patient continued to receive intraventricular chemotherapy (administered via the Ommaya reservoir), consisting of cytarabine (30 mg; 2 ×/week) alternating weekly with etoposide (0.5 mg; 5 ×/week; once daily) and topotecan (0.4 mg; 2×/week), based on our centers experience regarding intraventricular chemotherapy for disseminated brain tumors in the pediatric population [22]. In general, the therapy was well tolerated by the patient; merely the dosage of everolimus had to be adjusted continuously according to target serum levels, as well as painful aphthous ulcers in the mouth, ten weeks after initiation of therapy. During the course of therapy, the cell count and protein value in the CSF normalized and five weeks after initiation of intraventricular chemotherapy no malignant cells were detected in the CSF (Figure 1A).

**Figure 3:**
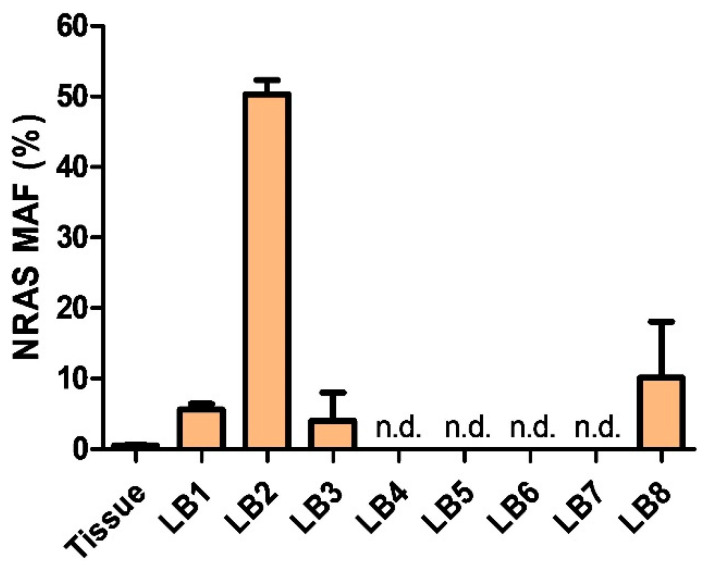
Liquid biopsy shows the mutant allele frequency (MAF) of mutant *NRAS* in tumor tissue (mean of 2 experiments) and in CSF liquid biopsy (mean of 5 experiments each). LB4-LB7 were negative for *NRAS(Q61R)* and NRAS-wt. LB 8 showed an increase of *NRAS(Q61R)*. Additional information given in Supplementary Materials Figure S2.

To investigate the underlying molecular alteration for a potential personalized therapy, we performed panel sequencing with Oncomine Comprehensive Assay v3 (Thermo Fisher Scientific, Waltham, MA, USA) in both tumor tissue biopsy and CSF liquid biopsy, revealing an underlying *NRAS(Q61R)* mutation. Interestingly, the mutant allelic frequency was higher in liquid biopsy (3%) as compared to the biopsy tissue (1%). These data suggest that liquid biopsy might be more feasible for tumor DNA analyses in this diffuse leptomeningeal tumor type with close connection to the CSF (Figure 3). The patient could be discharged and continue his activities of daily living, including proceeding with his education and participating in school via distance learning.

Three months after diagnosis, the MRI scan (Figure 1B, M3) showed a mixed response in terms of partial regression of some lesions, while others were progressive and newly detected. Based on the experience with *NRAS*-mutated melanomas, we initiated therapy with a checkpoint-inhibitor. Therefore, the therapy with everolimus was discontinued, and intravenous therapy with PD-1 inhibitor nivolumab (3 mg/kg, initially every two weeks, later every three weeks) was initiated. The main side effects experienced by the patient were gastrointestinal symptoms, which were managed well with steroids. 

After two administrations of nivolumab, an MRI was performed, again revealing a mixed response (Figure 1B, M4). Considering the experience with immune therapy in MM, trametinib was discontinued, and ipilimumab (2 mg/kg, every three weeks), a CTLA-4 inhibitor, initiated, the latter being reported to be effective in combination with nivolumab in metastasized MM [23]. 

This combination of treatment initially led to marked clinical improvement and normalized CSF parameters without detection of tumor cells in the CSF four months after initial diagnosis. Furthermore, this therapy could safely be administered in an outpatient setting, thereby substantially improving quality of life. During the further course of treatment, paresthesia of the lower extremities and ataxia warranted additional radiological evaluation. The subsequently performed MRI scan revealed further progression of multiple intracranial, as well as spinal tumorous lesions (Figure 1A, M5). Due to myelocompression of the thoracic spinal cord (Th 5–6), focal radiotherapy was initiated, which provided symptomatic relief, however, only for a brief period of time. Shortly thereafter, due to clinical deterioration, including right sided hemiparesis and reduced vigilance, the patient was admitted to the neuro-oncology ward. In view of renewed massive tumor progression on MRI scans (Figure 1A, M6) and the worsening clinical state of the patient, it was agreed with the patient and his family to discontinue oncologic treatment. The patient was released into a palliative care setting at home, where the patient succumbed to his disease two weeks after his discharge and 7 months after initial diagnosis.

### 3.2. Clinical Characteristics of PDLMM

**Figure 4:**
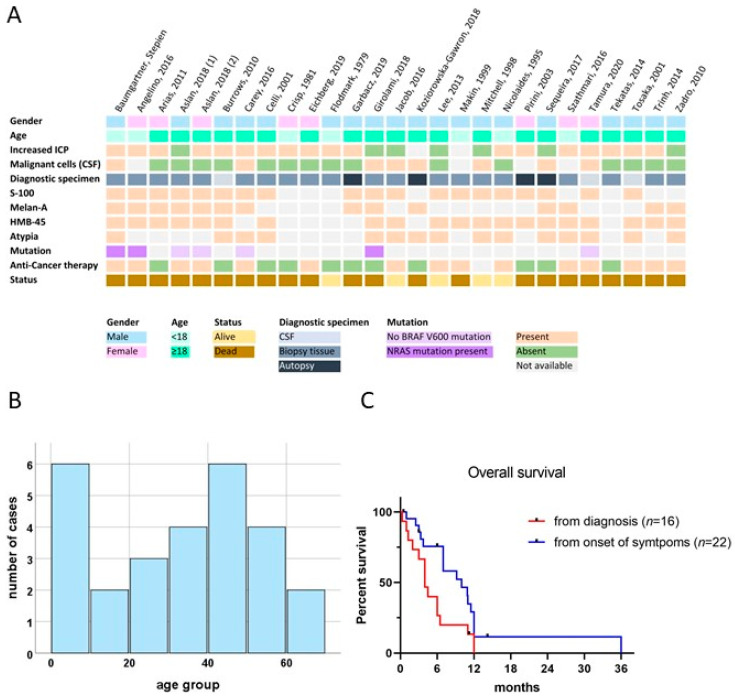
Results of the comprehensive literature review, including survival data and demographic properties. (**A**): Integrative overview of demographics, clinical, cytological, histological, and molecular findings of cases of PDLMM reported in literature, including our illustrative case. (**B**): Age histogram showing the bimodal age distribution. (**C**): Kaplan Meier curve for survival from diagnosis (red) and from onset of symptoms (blue). Patients alive at time of reporting were censored at latest mentioned time point.

We identified 26 cases [9,10,11,12,13,24,25,26,27,28,29,30,31,32,33,34,35,36,37,38,39,40,41,42,43] by screening EMBASE and PubMed for leptomeningeal melanomatosis, which provided sufficient information to be reliably classified as PDLMM. The main reason for exclusion were nodular lesions suggestive of primary CNS melanoma, or low mitotic frequency and lack of atypia and invasion signs indicative of primary leptomeningeal melanocytosis (Supplementary Materials Figure S1). An integrative overview of the analyzed literature, including our case, is depicted in Figure 4A (and Supplementary Materials Table S1).

Despite of this relatively low number, the analysis of the available data highlighted the following clinical properties, which are summarized in Table 1.

There was a male preponderance in PDLMM with a M:F ratio of 19:8, three each male and female patients were below the age of 10 years at diagnosis. The age distribution was bimodal, with a first peak in preschool children and a second peak in the 5th decade of life. The median age at diagnosis was 36 years (Table 1; Figure 4B).

The most common symptoms were related to increased intracranial pressure. More than half of all the patients analyzed (56%) presented with headaches, nausea, and vomiting (Table 1). Other clinical symptoms included seizures, cranial nerve palsy, ataxia, cognitive decline, memory loss, and mental status changes. Our patient initially presented with headaches, nausea, and vomiting albeit without clear signs of increased intracranial pressure on MRI (Figure 1; M1). More than half (*n* = 15) of the reported cases required an intervention to reduce ICP. As described in our case, cytologic analysis of CSF is important for early diagnosis. Malignant cells were found in 7 (33%) of reported patients but were also missing in two-thirds of patients where CSF was analyzed (14 cases). Other typical findings were elevated protein (67%), low glucose (76%), and pleocytosis (86%) (Table 1).

Although some cases were only diagnosed by autopsy (*n* = 4), the majority of the diagnoses were made by tissue sample analysis from surgical biopsies (*n* = 20). In three cases, diagnosis was solely based on CSF cytology. Typical markers used for diagnosis were S-100, HMB-45, and Melan-A. Only 11 studies reported on Ki67 or MIB1, which ranged between three and over ten percent, with some authors only reporting on a high Ki67 level. 

In the evaluated cohort of PDLMM patients, including our case, only six tumor samples were analyzed for *BRAF* mutation, which were all negative. Three were profiled in more detail, and two of them showed an *NRAS(Q61K)* mutation, similar to the mutation detected in our case *NRAS(Q61R)* (Figure 4A). 

Only about half of the patients received anticancer therapy and treatment strategies varied widely (Figure 4A; Supplementary Materials Table S1). The most common therapeutic approach was chemotherapy (*n* = 10), followed by immunotherapy (*n* = 5) and radiotherapy (*n* = 5). Longest survival was reported in one infant being treated with irradiation, chemotherapy, and pegylated interferon alpha (11 months, case 2, Supplementary Materials Table S1), as well as one young adult being treated with temozolomide (12 months, case 4, Supplementary Materials Table S1). Based on the available data, no clear benefit of a specific therapeutic approach could be concluded.

Analysis of survival proportions underlined that PDLMM has a dismal prognosis, with a median overall survival from diagnosis of only 4 months (range 0–12 months) and no survival exceeding 12 months in our pooled analysis (Figure 4C). Overall survival from symptom onset, however, may be substantially longer (median 10 months, range: 0.5–36) because of the frequently delayed diagnosis in this complex disease. 

### 3.3. Detection of NRAS-Mutant cfDNA via Liquid Biopsy

Since radiologic findings are often unspecific, and especially radio- and immunotherapy may also cause treatment related changes in imaging, assessment of therapy effectiveness is challenging. Based on the detection of cfDNA in CSF, we applied longitudinal testing of *NRAS* mutant allele frequency. Using digital droplet PCR (ddPCR), we were able to identify the characteristic *NRAS* mutation in the CSF samples of our patient. ddPCR allows for quantification of allele frequency, thereby potentially enabling disease monitoring. In line with results from NGS, the mutant allele frequency (MAF) in tissue was only 0.5% despite good detection of *NRAS*-wt (Supplementary Materials Figure S2). In contrast, and again corroborating results from NGS, MAF in liquid biopsy was 6% at the time of biopsy (Figure 1A and Figure 3, LB1). During the further course, MAF drastically increased peaking at 50% just before start of intraventricular therapy (Figure 1A and Figure 3 LB2). Subsequently, MAF dropped again to 4% following one month of intraventricular therapy, and PCR became negative for *NRAS*-mt eleven weeks after diagnosis (Figure 1A and Figure 3; LB4–6). Finally, MAF increased again at the time of tumor progression (Figure 1A and Figure 3, LB8) to 10%, underlining the potential of liquid biopsy as a tool for disease monitoring. However, disease progression could not be detected via liquid biopsy at initial progression (Figure 1A and Figure 3, LB7), and further studies to determine the sensitivity are warranted.

### 3.4. In Vitro Drug Sensitivity and Pathway Activation in Patient Derived PDLMM Cells

**Figure 5:**
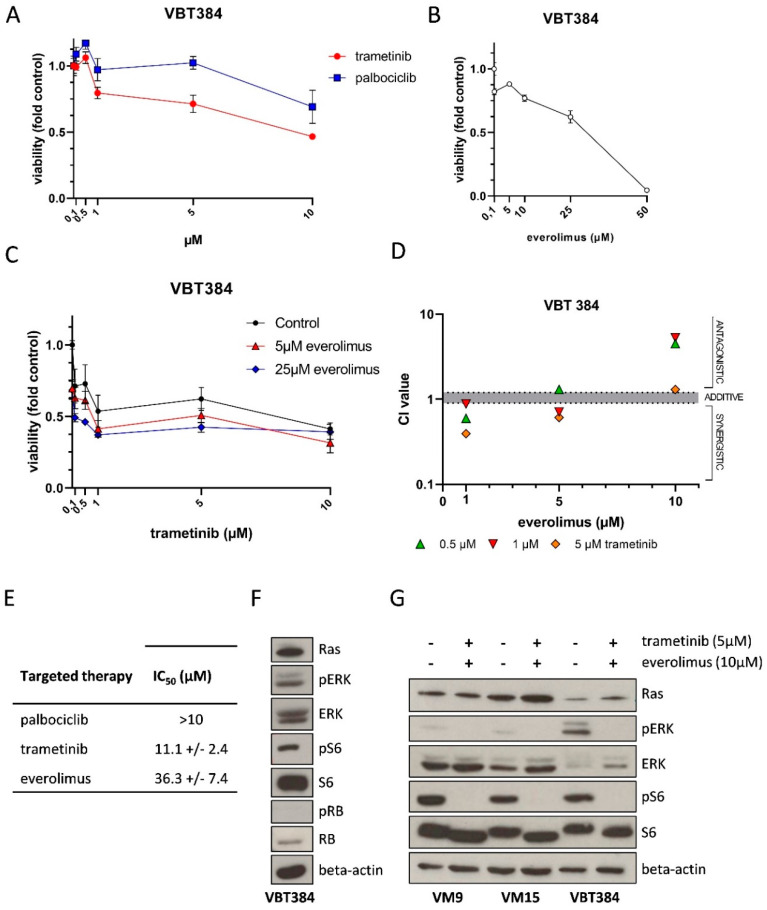
Sensitivity of patient-derived PDLMM cells to anti-cancer compounds and pathway activation. VBT384 cells were exposed to trametinib, palbociclib (**A**), everolimus (**B**) or indicated combinations (**C**) in the indicated concentrations for 72 h. Cell viability was measured by Cell-titer glo assay. Depicted results represent triplicates. Combination indexes from the experiment indicated in C were calculated according to the Chou-Talay method (**D**). IC_50_ values calculated for palbociclib, trametinib and everolimus in VBT384 cells (**E**). Western blot for detection of basal activation of mitogen-activated kinase (MAPK), PI3K, and RB-signaling in VBT384 cells as indicated (**F**). Western blot of melanoma cell models (VM9, VM15) and patient-derived VBT384 cells for indicated proteins and phosphorylations upon trametinib and everolimus combination treatment for six hours before protein isolation (**G**).

To further evaluate the treatment options derived from MM in PDLMM, we established a cell line (VBT384) of the biopsied tumor tissue. Due to the activation of the MAPK– and PI3K pathway, we tested the effect of trametinib and everolimus in the VBT384 cell model, recapitulating our treatment of the respective case. Under standard culture conditions, treatment with increasing concentrations of palbociclib, everolimus, and trametinib resulted in a dose-dependent decrease in cell viability (Figure 5A,B). However, VBT384 IC_50_ values above 10 µM were clearly exceeding clinically achievable doses in the short-term exposure (72 h), as depicted in Figure 5E. Combined application of trametinib and everolimus was tested in short-term exposure with ATP assay. Intriguingly, MEK inhibition distinctly synergized with everolimus at low concentrations of 1 µM in the VBT384 cell line (Figure 5C,D), however, still lacking a strong cytotoxic effect. We further explored activation of oncogenic pathways in VBT384 cells and identified phosphorylation of both Mitogen-Activated Protein Kinase 1 (ERK) and S6, in line with activation of MAPK and PI3K signaling (Figure 5F). Moreover, we detected no phosphorylation of Retinoblastoma-associated protein (Rb), indicating no aberrant activation of cyclin dependent kinases 4 and 6 (CDK4/6) as frequently found in cells harboring *CDKN2A* deletion. In addition, we compared this model to established *NRAS* mutant melanoma models (VM9 and VM15). Interestingly, NRAS levels were markedly lower in VBT384 still showing comparable levels of phosphorylated S6 thus confirming activation of the PI3K signaling cascade (Figure 5G). Notably, MAPK activation indicated by ERK phosphorylation was highest in the PDLMM cell model. Combination treatment with trametinib and everolimus effectively inhibited both ERK and S6 phosphorylation across all models, confirming the impact of these inhibitors on their respective targets (Figure 5G). 

## 4. Discussion

Leptomeningeal melanomatosis is a rare variant of a primary melanocytic tumor of the CNS, further characterized by a diffusely invasive leptomeningeal growth pattern. Diagnosis requires high suspicion by the treating physicians because radiologic findings and clinical symptoms can often be unspecific. Our comprehensive analysis of 27 cases reveals a fatal prognosis of only 4 months median overall survival following diagnosis. However, median overall survival after onset of symptoms is substantially higher (10.0 months) resulting from a long prediagnostic interval with a median of three months from symptom onset to diagnosis. This delay in diagnosis may worsen the already unfavorable prognosis. Increased intracranial pressure is a common symptom of PDLMM potentially due to mass effect of tumor and even more invasion of tumor cells into the leptomeninges and subsequent interference of cerebrovascular circulation. Our analysis revealed that this is a very common obstacle in diagnosis of PDLMM, as radiographic findings are often unspecific at time of diagnosis although 67% of patients suffer from increased intracranial pressure. Furthermore, by alleviating symptoms of ICP, such as headache, nausea, and vomiting, and showing increased opening pressure, lumbar puncture may be the first diagnostic clue regarding hydrocephalus. Consequently, it needs to be emphasized that absence of specific radiological findings, amongst other dilated ventricles on neuroimaging, cannot exclude ICP and warrant additional evaluation. We discovered that 37% of patients were in need of ventriculo-peritoneal shunt (VP-shunt) implantation, and another 19% necessitated other alleviating methods for increased ICP. If imaging is negative or increased intracranial pressure is suspected regardless of imaging findings, early-onset lumbar puncture and subsequent analysis of CSF and liquid biopsy are essential. Classic CSF findings of PDLMM include high protein concentration, resulting from blood-brain-barrier breakdown and concomitant protein production by tumor cells, as well as low glucose concentration, due to increased metabolism of tumor cells and impaired transport. Reactive lymphocytic pleocytosis and cytologic identification of malignant cells are additional typical CSF findings in PDLMM. Not all of these features need to be present to make the diagnosis, but an entirely normal CSF analysis is very uncommon. Nevertheless, as reported in several cases [12,43], malignant cells may only be detected in the course of repeated lumbar punctures, therefore the absence of malignant cells in primary CSF analysis does not exclude PDLMM as a possible diagnosis. In near future, an additional diagnostic tool may be liquid biopsies, allowing to screen for mutations without the need for identifying a tumor bulk. The present case is an excellent example for how liquid biopsy may be even more sensitive than tissue biopsy for mutation detection, as it is shown by the higher mutation allele frequency encountered in CSF compared to tumor tissue (Figure 3). Therefore, liquid biopsy is an important extension of classical cytology, especially when it comes to identifying targets for personalized treatment. Moreover, as PDLMM patients frequently require implantation of a VP-shunt for management of increased ICP liquid biopsy could be additionally used for longitudinal monitoring of disease, as shown in our report.

Despite the possibility of CSF analysis, histopathological analysis of tumor tissue is an essential component of establishing the diagnosis of PDLMM. The diffuse growth pattern constitutes a limiting factor in acquiring representative and sufficient tissue samples, leading to false negative biopsies in some cases [9,10]. It is, therefore, inevitable to identify the optimal site for biopsy beforehand using adequate imaging technologies. Classical imaging modalities used for diagnosis include CT and MRI, however, PET imaging brings the additional benefit of detecting (neoplastic) activity, thereby excluding systemic disease (FDG-PET), and allowing to establish the appropriate biopsy site (Methionine-PET). As an alternative to PET-CT, exclusion of MM can also be done by a careful examination of skin and eyes, as well as endoscopy of the gastrointestinal tract. On CT scans, melanocytic tumors appear as hyper-dense lesions, which are typically enhanced after the injection of gadolinium, therefore mimicking hemorrhage. MRI scans provide more detailed diagnostic information, typically showing hyperintensity of lesions on T1-weighted magnetic resonance and iso- to hypointensity on T2-weighted magnetic resonance images (Figure 2A). However, these findings are not always present, as shown in our case. Radiological findings may also be misleading, mimicking tubercular meningitis, highlighted by six (23%) patients being initially treated for tuberculosis, and other infectious, vascular and neoplastic diseases. 

As mentioned initially, conventional chemotherapy is neither effective in PDLMM nor in MM with leptomeningeal CNS involvement. Within a comprehensive review of MM patients with leptomeningeal melanomatosis the only positive predictive factor is the application of intraventricular therapy [44]. Besides our patient, only one author reported the use of intraventricular therapy in PDLMM which resulted in an overall survival of 12 months [12]. Based on our centers experience with intraventricular therapy [22,45,46], we support the application of cytotoxic drugs directly into CSF, thereby reaching higher concentrations in the leptomeningeal malignancy and adjacent affected tissue. The continuous application of intraventricular therapy with alternating cytotoxic agents led to rapid decrease of malignant cells in CSF and significant clinical improvement of our patient.

To further elucidate the molecular background of PDLMM, we analyzed the tumor tissue, as well as cfDNA, obtained from the CSF samples from our patient, and found an *NRAS(Q61R)* mutation. This finding was in line with results from our literature review, which revealed that all PDLMM cases with mutational data available harbored a *NRAS* mutation (*n* = 3). Moreover, three additional cases were only tested for *BRAFV600E* mutation, which was negative, suggesting another oncogenic driver mutation. These data support that PDLMM not only emerges from the same precursor cell but also shares molecular characteristics with MM, therefore supporting the application of novel MM therapies to PDLMM. We compared these data with reports on PDLMC (histologically, the closest entity to PDLMM), which is characterized by diffuse proliferation of melanocytes without invasion of the CNS parenchyma, often associated with neurocutaneous melanosis, defined by multiple congenital giant melanocytic naevi and primary benign melanocytosis of the leptomeninges. Neurocutaneous melanosis is not inherited in a classical Mendelian way, however, since diverse cutaneous and intracranial lesions from the same individual were shown to harbor the same mutation the hypothesis established, that the disease develops after a single postzygotic mutation in a melanocytic precursor cell [47]. In a previous study, 10 out of 13 cutaneous samples showed an *NRAS* mutation on Codon 61 with a predominance of the c.181C > A, *p.Q61K* mutation over the c.182A > G, *p.Q61R* [48]. Similar, an *NRAS(Q61K/R)* mutation was found in 75% of their patients [49]. Analysis of the remaining samples for *BRAF* mutations revealed that 12.5% were positive for *BRAFV600E* and 12.5% did not harbor either mutation [48]. Only ten cases [50,51,52,53,54,55,56,57,58] of isolated leptomeningeal melanocytosis are well documented in the literature, and some authors refer to these cases as “forme frustre” of neurocutaneous melanosis, i.e., neurocutaneous melanosis without cutaneous manifestations. Molecular data is only available for two of these cases, showing, again, lack of *BRAF* mutation in one [57] and mutated *NRAS* in the other case [58]. 

With respect to treatment modalities, two main groups of innovative therapies have been established for MM treatment. First, immunotherapies, in particular checkpoint inhibitors and second, inhibitors of the RAS-BRAF-MAPK signaling pathway. These therapies extended the one-year overall survival of patients with MM from 25% to almost 85% [59].

It has been found that the high mutational load characterizes melanoma as especially immunogenic. Nevertheless, T-cell exhaustion and anergy are also present in the melanoma microenvironment [60], making it an interesting target for immune checkpoint inhibitors. These inhibitors are directed against either CTLA-4 (i.e., ipilimumab), thereby enhancing T-effector cells and inhibiting T-regulatory cells during the activation phase, or against PD-1 (i.e., nivolumab, pembrolizumab), a receptor on the surface of activated lymphocytes, responsible for T-cell exhaustion [60]. Initial studies with ipilimumab alone already showed an increased long-term survival of 20%. The combination of nivolumab and ipilimumab revealed superiority with a 5-year survival of 52% [23]. Especially for *NRAS*, mutant melanoma combination therapy is essential [61]. Patients with metastases to the brain also benefitted from the combination therapy, with an intracranial response in 46% of patients who received the combination and 20% intracranial response rate for the single agent nivolumab, irrespective of their mutational status [59,62,63].

For patients with *BRAF*-mutated MM without any response to immunotherapy or with recurrence, the combination of BRAF- and MEK-inhibitors is a promising alternative, as was shown in a recent study on binimetinib [16]. The combination showed superiority over the single agent therapy, not only in response rates, 70% versus 50%, respectively, but also in progression-free survival (PFS; HR 0.70, 95% CI 0.59 to 0.82). Further, a paradoxical activation of the MAPK pathway in BRAF single-agent inhibition is contained by the combination therapy [4,64]. The role of this novel therapy in patients with brain metastases was long unexplored since many studies excluded patients with intracranial metastasis. Finally, studies that included these patients showed an intracranial disease control rate of 60–79% and a median overall survival of 9.5 and 11.2 months, proving that targeted therapy has an effect despite the blood brain barrier [65]. However, all this data is accurate for *BRAFV600E* mutated tumors, but not for melanoma with an *NRAS* mutation. Since *NRAS* activates multiple downstream pathways, single agent inhibition with MEK inhibitors was not sufficient to improve overall survival when compared to chemotherapy [66] and *BRAF* inhibition results in a paradox RAF activation. We could confirm this effect in vitro using our unique patient derived cell model demonstrating single-agent effects of trametinib and everolimus, however, outside the range of clinically achievable doses. Nevertheless, a promising option is the combination of MEK inhibitors with inhibitors of the PI3K pathway, thereby blocking two key signaling pathways of NRAS [67], which has already been shown for *NRAS*-mutant MM. Investigation of respective downstream signaling in our study confirmed efficient block of MAPK and PI3K-signaling in PDLMM mirroring the effects in *NRAS*-mutant MM cell models. Corroboratively, the drug combination of trametinib and everolimus showed a synergistic effect thus supporting the application also in *NRAS* mutated tumors. However, Phase I studies for melanoma showed considerable side effects of this drug combination, which limited the drug exposure [66,68]. In our experience with combination of targeted treatment, also including everolimus and trametinib, the side effects were manageable for a certain period in pediatric patients, but combination therapy had to be discontinued eventually due to skin toxicity [69]. Nevertheless, we initiated this combination of treatment in the reported case, due to urgent clinical need. This decision was further supported by a case series on primary CNS melanoma in pediatric patients, where MEK inhibition led to symptom stabilization [70]. Unfortunately, the MRI three months after diagnosis (Figure 1B, M3) showed a mixed response leading to the evaluation of other treatment options.

A known obstacle in evaluation of treatment response during therapy with immune checkpoint inhibitors is the so-called pseudoprogression in radiologic imaging [71]. Liquid biopsies may be a helpful tool for distinguishing between tumor progression and radiologic pseudo-progression and therapy monitoring in general. This novel technique is already under intensive investigation for routine monitoring and therapy management of both MM and brain tumor patients [72,73,74,75,76]. The continuously collected CSF samples during the course of treatment enabled us to utilize CSF for liquid biopsy. As CSF circulating through the CNS is in close contact with intracranial lesions, liquid biopsy of CSF has been described as a more sensitive method for detection of intracranial tumor mutations compared to liquid biopsy of plasma [77,78]. Our serial analysis of CSF samples for *NRAS* mutation using ddPCR showed a correlation between tumor cells in the CSF cytology and amount of detected *NRAS.* Moreover, tumor-derived DNA was again detectable upon tumor recurrence. It remains to be determined if these findings can be replicated in other patients with PDLMM. 

## 5. Conclusions

We report the first case of a pediatric patient with PDLMM who received combination treatment including intraventricular chemotherapy (alternating between etoposide, topotecan and cytarabine) with concomitant trametinib, everolimus, and consecutively intravenous nivolumab, ipilimumab, and palliative irradiation, resulting in transient clinical improvement. This therapy regimen was, so far, administered mainly in an outpatient setting, thereby improving quality of life. 

Further studies including the use of liquid biopsies and preclinical models are necessary to develop new therapies improving treatment and prognosis of this rare and extremely aggressive disease.

## Figures and Tables

**Table 1:** Patient characteristics.

Characteristics		
Sex	*n*	%
Male	19	70
Female	8	30
Age	*n*	%
0 to 10	6	22
10 to 20	2	7
20 to 30	3	11
30 to 40	4	15
40 to 50	6	22
50 to 60	4	15
>60	2	7
		years
Median age		36
Age range		2.3–68
Symptoms	*n*	%
Nausea/vomiting	20	77
Headache	18	69
Cognitive deficits	11	42
Paresthesia	7	27
Cranial nerve palsy	6	23
Anorexia	6	23
Seizures	5	19
Amnesia	5	19
Peripheral paresis/plegia	4	15
Ataxia	3	12
Fever	3	12
Any pain	2	7
Fatigue	1	4
Increased intracranial pressure	*n*	%
Yes	18	67
No	7	26
Unknown	2	7
Intervention for increased ICP	*n*	%
Yes	15	56
No	12	44
CSF values	*n*	%
Pleocytosis	18	86
Elevated protein	14	67
Low glucose	16	76
Malignant cells in CSF	*n*	%
Yes	7	33
No	14	67
Anti-tumor therapy	*n*	%
Irradiation	5	20
Chemotherapy	10	40
Targeted therapy	1	4
Immunotherapy	5	20
Intraventricular therapy	2	8
Anti-tumor therapy (total)	14	56

## Supplementary Materials

The following are available online at https://www.mdpi.com/article/10.3390/jpm11040292/s1, Supplementary Figure S1: Prisma flow diagram depicting citations identified, screened and included in the comprehensive literature review. Supplementary Figure S2: Scatter plots of ddPCR analysis of CSF derived ctDNA for liquid biopsy detection of NRAS(Q61R) and NRAS-wt. Supplementary Table S1: demographic and clinical data derived from the case reports included in our comprehensive literature review. Supplementary Table S2: Antibodies used for Western Blot.
