# Supplementary material for: Novel Insights into Diagnosis, Biology, and Treatment of Primary Diffuse Leptomeningeal Melanomatosis
Source: J Pers Med. 2021 Apr 12;11(4):292. doi: 10.3390/jpm11040292 (PMC8069125; doi:10.3390/jpm11040292)
Supplement: Supplementary file 1 [file jpm-11-00292-s001.pdf]

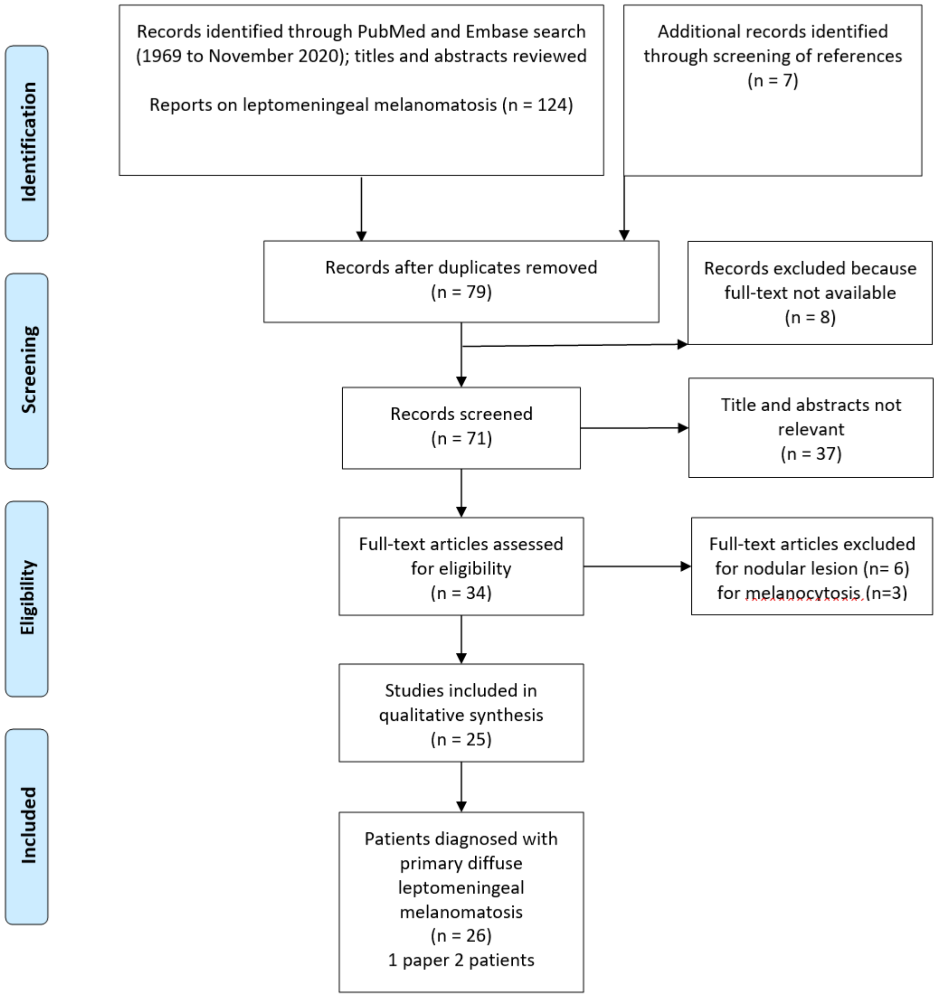

Supplementary Figure 1: Prisma flow diagram depicting citations identified, screened and included in the comprehensive literature review. Template from Moher et al., 2009.

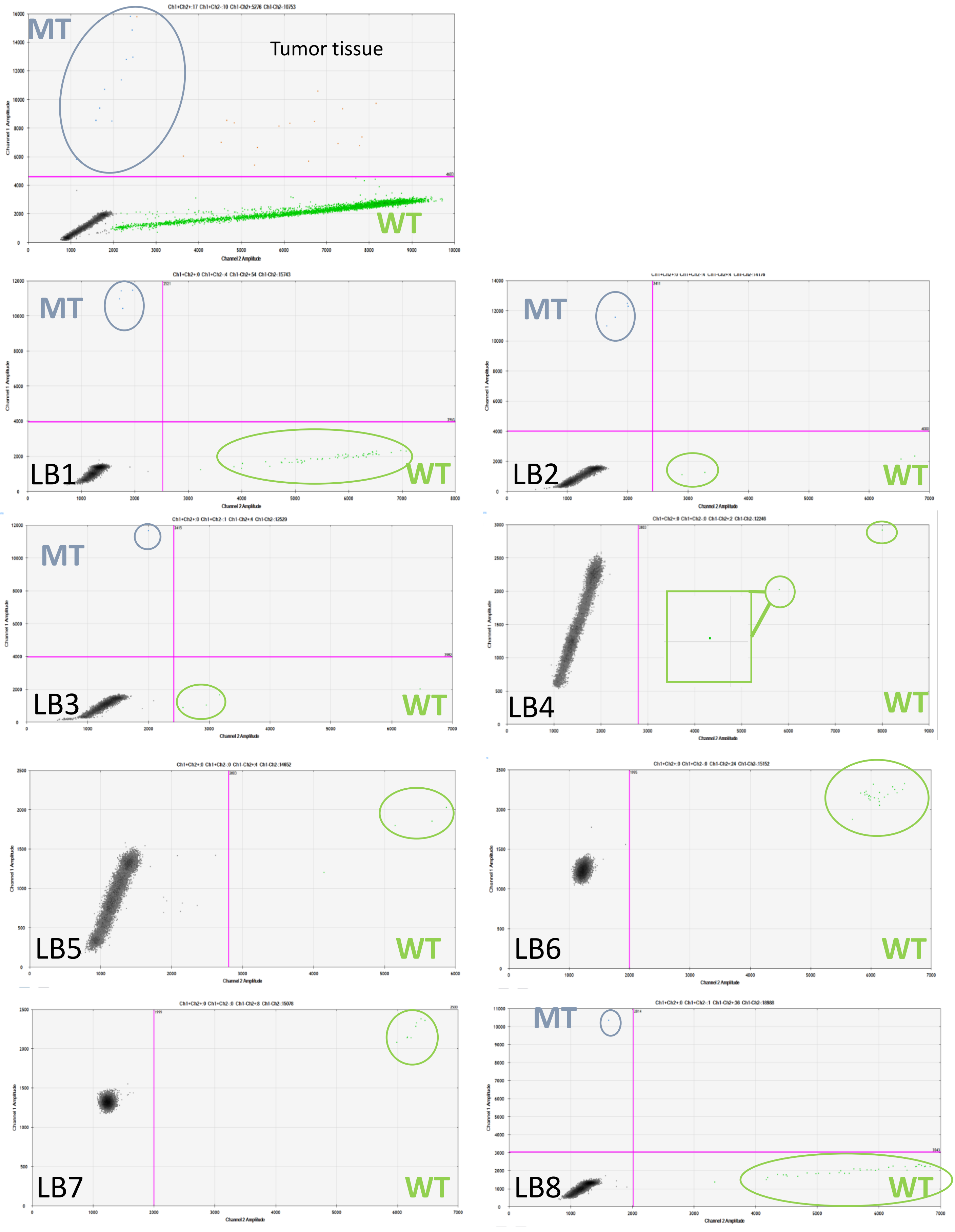

Supplementary Figure 2: Scatter plots of ddPCR analysis of CSF derived ctDNA for liquid biopsy detection of *NRAS(Q61R)* and *NRAS-wt*. MT = *NRAS(Q61R)*, WT= wild type, LB1-8 = different time points as indicated in Fig1A.

Author, year	Case #	Sex	Age (years)	time to diagnosis (months)	Histological diagnosis	Treatment	survival from diagnosis (months)
Baumgartner/Stepien, 2020	1	m	15	0.5	Biopsy	Trametinib, Everolimus; i.th. therapy, Nivolumab, Ipilimumab, Irradiation	6.5
Angelino, 2016	2	f	2.3	>0.5*	Biopsy	Irradiation; TMZ, Cisplatin, Vindesine; PEG-INF alpha-2-B;	11
Arias, 2011	3	f	40	-	Biopsy	no therapy	-
Aslan, 2018	4	m	21	-	Biopsy	TMZ	12
Aslan, 2018	5	f	44	-	Biopsy	Irradiation; Cisplatin, Dacarbazine	4
Burrows, 2009	6	m	32	1.2	CSF	no therapy	1.3
Carey, 2016	7	m	36	6	Biopsy	Ipilimumab	1
Celli, 2001	8	m	53	11	Biopsy	no therapy	1
Crisp, 1981	9	f	4.5	3	Biopsy	no therapy	-
Eichberg, 2019	10	f	44	7	Biopsy	Immunetherapy	4
Flodmark, 1979	11	m	3	0.5	Biopsy	no therapy	-
Garbacz, 2019	12	m	45	7	Autopsy	no therapy	0
Girolami, 2018	13	m	38	3	Biopsy	no therapy	0.3
Jacob, 2016	14	m	55	>6*	Biopsy	TMZ	-
Koziorowska-Gawron, 2018	15	m	68	-	Autopsy	no therapy	0
Lee, 2013	16	m	17	-	Biopsy	Irradiation; Cisplatin, BCNU Tamoxifen, Dacarbazine,	-
Makin, 1999	17	m	5.5	3.2	Biopsy	Carboplatine, Etoposide, VCR	6
Mitchell, 1998	18	m	49	3	Biopsy	not reported	
Nicolaides, 1995	19	m	5	4	Biopsy	Chemotherapy (local PNET protocol)	
Pirini, 2003	20	f	68	36	Autopsy	no therapy	0
Sequeira, 2017	21	m	53	1	Autopsy	no therapy	0
Szathmari, 2016	22	f	5	4.9	Biopsy	Etoposide, TMZ Carboplatin,	24
Tamura, 2020	23	f	49	3°	3rd CSF	Nivolumab, Ipilimumab	4
Tekatas, 2013	24	m	21	8	2nd Biopsy	no therapy	2
Tosaka, 2001	25	m	20	7.5	2nd CSF	Dacarbazine, ACNU, VCR; i.th. MTX	4.5
Trinh, 2014	26	m	51	-	2nd Biopsy	Irradiation; TMZ	
Zadro, 2010	27	m	34	-	Biopsy	Irradiation	

Supplementary Table 1: demographic and clinical data derived from the case reports included in our comprehensive literature review. M, male; f, female; CSF cerebrospinal fluid; i.th. intrathecal; TMZ temozolomide; PEG-INF alpha 2B: PEG-Interferon alpha 2B; BCNU ; VCR vincristine; PNET primitive neuroectodermal tumor; ACNU nimustine; MTX methotrexate. *1 a certain interval was given, afterwards it was not specified how much longer it took until final diagnosis. *2 self-extubation, did not succumb to the disease itself.

Antibody	Source
ERK 1/2 (p42/44 MAPK) (#9102)	Cell signaling, Danvers, MA, USA
P-ERK 1/2 (Thr202/Tyr204) (#9101)	Cell signaling
Rb (IF8 (#sc-102)	Santa Cruz Biotechnology, Dallas, TX, USA
P-Rb (Ser807/811) (#9308)	Cell signaling
S6 (5G10) (#2217)	Cell signaling
pS6 (ser240/244) (#5364)	Cell signaling
Ras (#610001)	BD Transduction Laboratories, San Jose, CA, USA
β-actin	Sigma-Aldrich, St.Louis, MO, USA

Supplementary Table 2: Antibodies used for Western Blot.
